# Ribosomal protein L24 mediates mammalian microRNA processing in an evolutionarily conserved manner

**DOI:** 10.1007/s00018-023-05088-w

**Published:** 2024-01-23

**Authors:** Yonat Tzur, Serafima Dubnov, Nimrod Madrer, Adi Bar, Bettina Nadorp, Nibha Mishra, Paul Heppenstall, Estelle R. Bennett, David S. Greenberg, Katarzyna Winek, Hermona Soreq

**Affiliations:** 1 The Silberman Institute of Life Sciences, The Hebrew University of Jerusalem, Edmond J. Safra Campus, 91904 Jerusalem, Israel; 2 The Edmond and Lily Safra Center of Brain Science, The Hebrew University of Jerusalem, Edmond J. Safra Campus Givat Ram, Jerusalem, Israel; 3 The International School for Advanced Studies, Trieste, Italy; 4 Leibniz Institute on Aging, Fritz Lipmann Institute, Beutenbergstraße 11, 07745 Jena, Germany; 5 Present Address: New York City, USA; 6 Present Address: Waltham, USA

**Keywords:** DDX5, Hsa-miR-608, miR processing, Pre-miRs, Pri-miRs, RPL24

## Abstract

To investigate the mechanism(s) underlying the expression of primate-specific microRNAs (miRs), we sought DNA regulatory elements and proteins mediating expression of the primate-specific hsa-miR-608 (miR-608), which is located in the SEMA4G gene and facilitates the cholinergic blockade of inflammation by targeting acetylcholinesterase mRNA. ‘Humanized’ mice carrying pre-miR-608 flanked by 250 bases of endogenous sequences inserted into the murine Sema4g gene successfully expressed miR-608. Moreover, by flanking miR-608 by shortened fragments of its human genome region we identified an active independent promoter within the 150 nucleotides 5′ to pre-miR-608, which elevated mature miR-608 levels by 100-fold in transfected mouse- and human-originated cells. This highlighted a regulatory role of the 5′ flank as enabling miR-608 expression. Moreover, pull-down of the 150-base 5′ sequence revealed its interaction with ribosomal protein L24 (RPL24), implicating an additional mechanism controlling miR-608 levels. Furthermore, RPL24 knockdown altered the expression of multiple miRs, and RPL24 immunoprecipitation indicated that up- or down-regulation of the mature miRs depended on whether their precursors bind RPL24 directly. Finally, further tests showed that RPL24 interacts directly with DDX5, a component of the large microprocessor complex, to inhibit miR processing. Our findings reveal that RPL24, which has previously been shown to play a role in miR processing in *Arabidopsis thaliana*, has a similar evolutionarily conserved function in miR biogenesis in mammals. We thus characterize a novel extra-ribosomal role of RPL24 in primate miR regulation.

**Graphical abstract:**

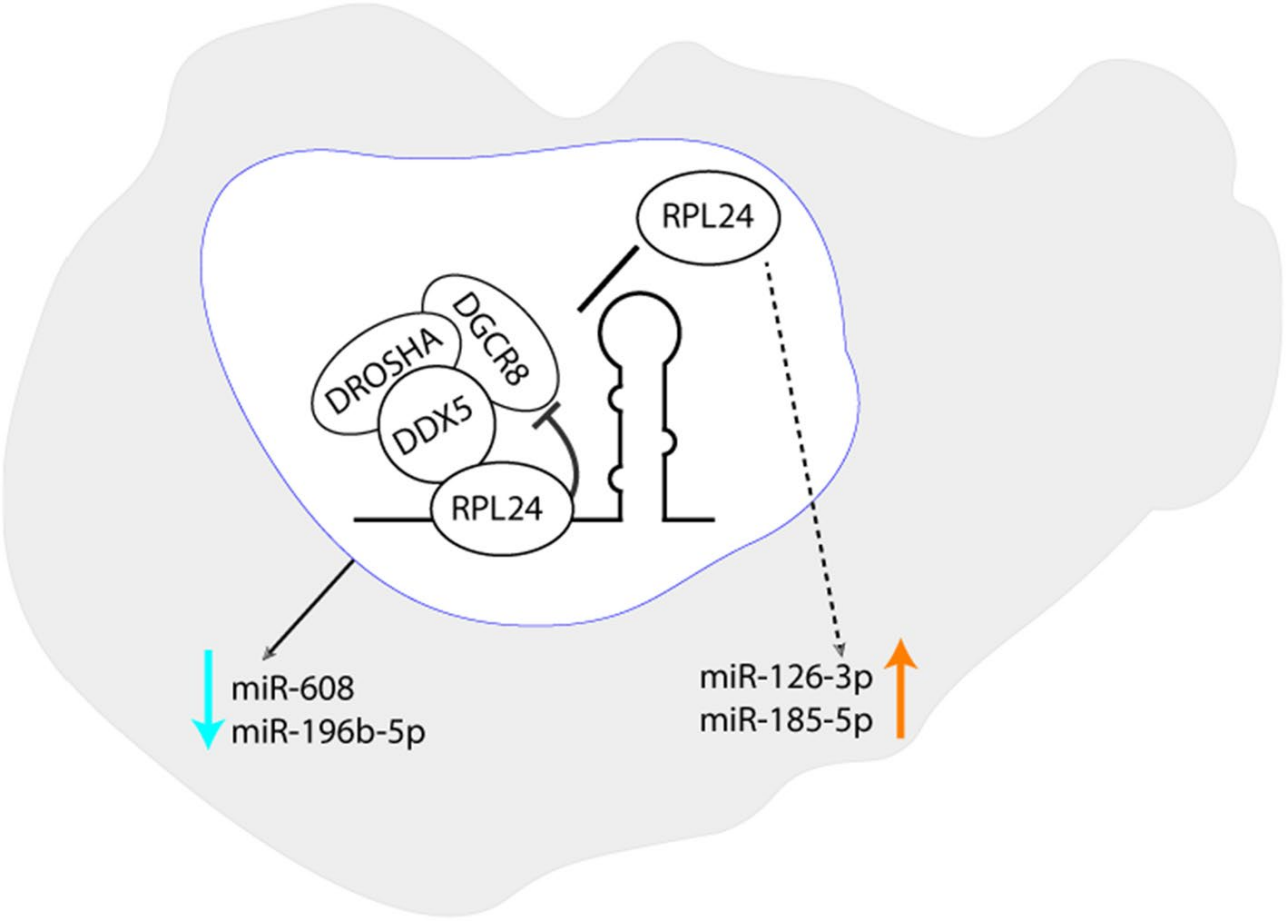

**Supplementary Information:**

The online version contains supplementary material available at 10.1007/s00018-023-05088-w.

## Introduction

Mammalian gene expression is modulated by microRNAs (miRs), small non-coding RNAs (~ 22 nucleotides long), which act by binding mRNA transcripts that contain complementary sequence motifs and silencing them post-transcriptionally [1]. Importantly, a large fraction of the known human miRs has been evolutionarily incorporated into the primate genome [2], but neither the mechanism(s) enabling their expression nor their global biological impact are known. In metazoans, RNA polymerase II transcribes the majority of primary miRs (pri-miRs), followed by processing in the microprocessor complex. This complex is comprised of the core components nuclear RNase III DROSHA and the double-stranded RNA-binding protein DGCR8, and includes additional auxiliary factors such as the RNA helicases DDX5 (p68) and DDX17 (p72) [3]. The resulting pre-miRs are transported to the cytoplasm by exportin 5 [4], where they are further processed by the RNase III Dicer. Mature miRs are then loaded onto a multi-protein RNA-induced silencing complex (RISC) that inhibits translation and/or promotes degradation of the target mRNA transcripts [5]. However, much remains unclear about the mechanism of microprocessor recognition and cleavage of pri-miRs.

The ‘basal’ junction between the single-stranded RNA and the double-stranded stem RNA of pre-miRs, and the size of the terminal loop of pri-miRs, are all crucial for microprocessor recognition and processing [6, 7]. In addition, several sequence elements such as the UG and CNNC motifs residing in the basal region of pri-miRs and the UGUG motif in the terminal loop are involved in pri-miR processing [8, 9]. However, only some pri-miRs carry these motifs, suggesting that other regulatory elements are involved as well. In this context we were interested in studying the primate specific hsa-miR-608, as it contains none of the motifs described above. This miR is located in the third intron of the primate SEMA4G gene, a member of the conserved Semaphorin family of proteins. Semaphorins participate in regulating nervous system development and activation of the immune response [10]. MiR-608 has been hypothesized to share a common promoter with its SEMA4G host gene [11, 12], but the precise mechanisms underlying its expression remained elusive.

In humans, miR-608 is expressed in several brain and peripheral tissues including forebrain, cerebellum, small intestine and liver (EMBL-EBI expression atlas; https://www.ebi.ac.uk/gxa/home). At the functional level, although expression is categorized in the atlas as “low”, miR-608 has been shown to target the acetylcholine hydrolyzing enzyme acetylcholinesterase (AChE), the inflammatory cytokine interleukin-6 (IL-6) and the cell division cycle 42 (CDC42) Rho GTPase, indicating relevance for the cholinergic blockade of inflammation [13] and reaction to stressful stimuli [14]. Interestingly, AChE recognition by miR-608 is interrupted by the clinically significant rs17228616 single-nucleotide polymorphism (SNP) found in the 3′-untranslated region (3′-UTR) of the AChE gene. Carriers of the minor, less abundant allele of this SNP show reduced miR-608 blockade of AChE, which results in a lower cholinergic tone [14]. This is accompanied by elevated blood pressure, increased inflammatory biomarkers, and prefrontal cortex blockade of amygdala stress reactions [14, 15]. In comparison, the rs4919510 SNP resides in the miR-608 gene itself and carriers of its minor allele show reduced miR-608 levels, limited miR-608 regulation of AChE and other targets, and protection from sepsis following head injury [16]. Given the importance of miR-608 contribution to the regulation of cholinergic tone, we sought regulatory elements that facilitate miR-608 expression and contribute to its impact on AChE expression and human health and well-being.

## Results

### Cis-sequences regulate miR-608 expression

**Fig. 1:**
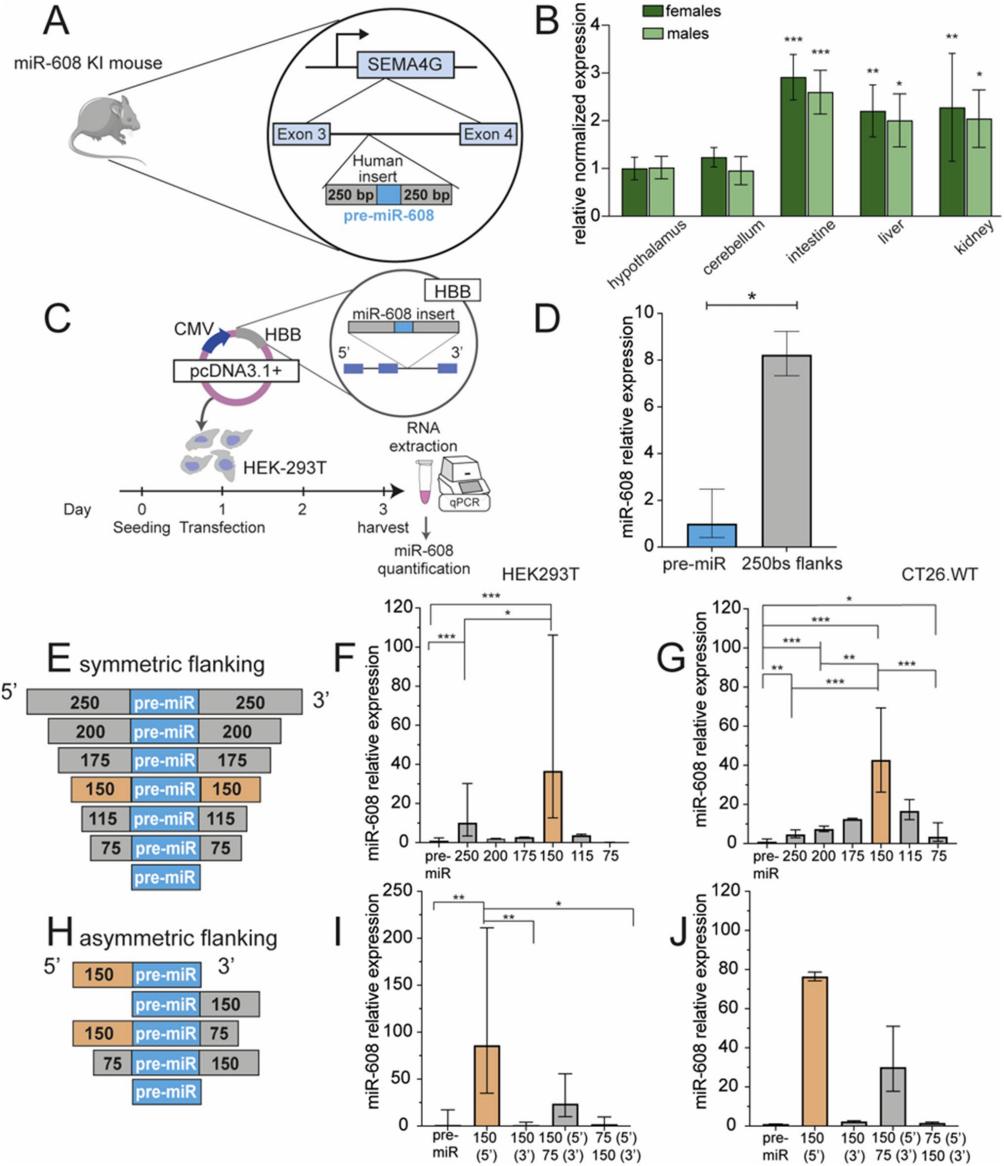
Cis-sequences regulate miR-608 expression in engineered KI mice and cultured cells. **A** Structure of the mouse construct: miR-608 transgenic ‘humanized’ mice were established by inserting hsa-pre-mir-608 into the third intron of the mouse Sema4g gene, with 250 flanking bases at each side. **B** miR-608 levels in the brain and peripheral tissues of female and male miR-608 KI mice, determined by RT-qPCR. All miR-608 levels were normalized to those of female hypothalamus (that showed the lowest expression); two-way ANOVA with Dunnet’s multiple comparisons correction, ± SD, *n* = 5 per group. **C** Experimental design: HEK293T and CT26.WT cells were seeded and 24 h later transfected with pcDNA3.1 + plasmid containing miR-608 inserted into the second intron of HBB and flanked by native sequences of varying lengths. Cells were harvested 48 h later, RNA extracted, and miR-608 levels quantified. **D** Symmetric bidirectional 250 base extension of the miR-608 stem-loop altered its levels in HEK293T cells; bar graph, ± SD, *p* = 0.0163, unpaired t-test. **E** Constructs of pre-miR-608 flanked by symmetric sequences ranging from 75 to 250 bases. **F** Levels of miR-608 expressed from these constructs in HEK293T cells. The 150 bases both upstream and downstream are critical for miR-608 expression in HEK293T cells. **G** The 150-base symmetrical flanks are also critical for expression in CT26.WT cells. **H** Constructs of pre-miR-608 flanked by asymmetric sequences ranging from 75 to 150 bases. **I** Levels of miR-608 expressed from these constructs in HEK293T cells were highest under control of the upstream 150 base sequence. **J** A similar effect is seen in CT26.WT cells transfected with these constructs. All experiments were performed in duplicate or triplicate and miR-608 levels were measured using Taqman RT-qPCR with RNU6B and snoRNA135 as normalizing genes. Results are shown relative to levels of pre-miR-608 with no flanking sequences. In all panels *p < 0.05, **p < 0.01, ***p < 0.001. In panels **F**, **G**, **I**, bar-graph ± SD, one-way ANOVA with Tukey’s multiple comparisons test

To explore and characterize the molecular regulation of miR-608 we aimed to engineer a ‘humanized’ mouse that expresses hsa-miR-608. To ascertain the relevance of this model we first assessed the predicted human targets potentially subjected to miR-608 regulation. Using the TargetScan 8.0 prediction tool [17], we identified 336 predicted targets (supp. Table 1). We then used the TargetScanHuman 5.2 Custom prediction tool [18] and identified 203 mouse targets (Supp. Table 2). We next determined that 52 (~ 25%) of the predicted mouse targets were also predicted in humans (Supp. Fig. 1A). Gene ontology (GO) analysis of these shared targets revealed enriched processes involving cell adhesion and brain development as potentially subjected to regulation by miR-608 (Supp. Fig. 1B). Based on the above, we established the ‘humanized’ hsa-miR-608 mouse. This involved inserting the pre-miR-608 sequence, flanked by 250 bases of intronic sequences from its human host gene SEMA4G, into the third intron of the mouse Sema4g gene (Fig. 1A). We quantified miR-608 levels in various brain areas as well as peripheral tissues from the miR-608 knock-in (KI) mice. This detected miR expression, albeit at low levels, in the hypothalamus and cerebellum as well as in the intestine, liver and kidney, with peripheral tissues showing higher levels than brain tissues. No difference was observed between female and male mice (Fig. 1B, Supp. Fig. 1C). In the hippocampus, medial-prefrontal cortex and heart miR-608 levels were below detection, possibly indicating some degree of tissue-specific regulated expression. Nevertheless, the transgenic mice presented a seemingly unchanged phenotype compared to control mice. No gross macroscopic changes were observed, and these mice maintained unchanged motor and anxiety traits as measured by rotarod, elevated plus maze and open field tests (Supp. Fig. 1 D–J). Their inflammatory profile, as measured by Tnfa and Nfkb levels was also unchanged (Supp. Fig. 1 K–L).

Next, we wished to determine if miR-608 expression, although low, had a functional effect in the KI mice. To this end, we quantified the mRNA levels of DnaJ (Hsp40) homolog subfamily B member 5 (DNAJB5), forkhead box P4 (FOXP4), and SIX homeobox 3 (SIX3), the top three predicted mouse targets of miR-608 that were also predicted in humans (Supp. Tables 1 and 2). No changes in Dnajb5 and Foxp4 were observed in any of the miR-608-expressing tissues in comparison to control mice (Supp. Fig. 2A–B), possibly due to the low expression levels of this miR. Further, we could not detect SIX3 in any of these tissues. To examine the impact of the inserted miR-608 on its host gene Sema4g, we quantified Sema4g mRNA levels in KI versus control mice. Interestingly, miR-608 KI mice presented elevated levels of Sema4g in liver, kidney, hypothalamus and cerebellum (Supp. Fig. 2C). Since miR-608 was thought to be transcribed from its host gene promoter [11, 12], we also expected the higher levels of Sema4g to lead to higher levels of miR-608. However, no significant correlation was found between Sema4g and miR-608 levels in the KI mice (Supp. Fig. 2D), suggesting that additional factors may be involved in regulating miR-608 expression.

Given that miR-608 is primate-specific, we predicted that its expression in a non-primate organism was facilitated by the flanking intronic sequences from its human host gene. To identify flanking sequences critical for miR-608 expression we created a series of constructs containing pre-miR-608 surrounded by different lengths of the native flanking bases. We then inserted these constructs into the second intron of the human hemoglobin subunit beta (HBB) gene that we cloned into the pcDNA3.1 + plasmid. We transfected both human (HEK293T) and murine (CT26.WT) cell lines with the various constructs and quantified mature miR-608 levels 48 h after transfection (Fig. 1C). Since basal miR-608 levels in HEK293T cells were below detection threshold (Supp. Fig. 3A) (and no basal expression is expected in the non-primate CT26.WT cells), any detected miR-608 could be attributed solely to the transfected constructs.

Transfecting cells with pre-miR-608 flanked by 250 bases at both the 5′ and 3′ ends resulted in miR-608 levels eightfold higher than in cells transfected with the pre-miR-608 lacking flanking sequences, indicating that the flanking sequences contain sufficient elements to enable miR-608 expression (Fig. 1D). To further define the regulatory sequences, we introduced symmetric flanks of gradually shortened lengths at both the 5′ and 3′ ends of pre-miR-608 and compared their expression efficacy to that of the pre-miR-608 construct with no flanking regions. Constructs with flanking sequences of 150 bases at both the 5′ and 3′ sides increased miR-608 expression by over ~ 40-fold in both HEK293T and CT26.WT cells (Fig. 1E–G). This indicated the presence of binding motifs for transcription activators within these sequences. In contrast, extending the symmetric flanking beyond those 150 bases greatly decreased miR-608 levels, possibly reflecting the presence of binding motifs for transcription suppressors in the sequences 150–250 bases upstream and/or downstream of pre-miR-608. To identify sequences indispensable for miR-608 expression, we expressed pre-miR-608 constructs with asymmetric or lacking flanks (Fig. 1H). Most asymmetric combinations of flanking sequences showed low expression in both cell lines, with the exception of the 5′ 150-base flanking sequence that was sufficient by itself to potentiate miR-608 expression by ~ 100-fold in HEK293T and ~ 80-fold in CT26.WT cells. Therefore, the absence of the 3′ sequence caused an increase of roughly twofold in miR-608 expression in both cell lines (Fig. 1H–J).

### The 5′ sequence includes a TATA box enabling miR-608 expression

**Fig. 2:**
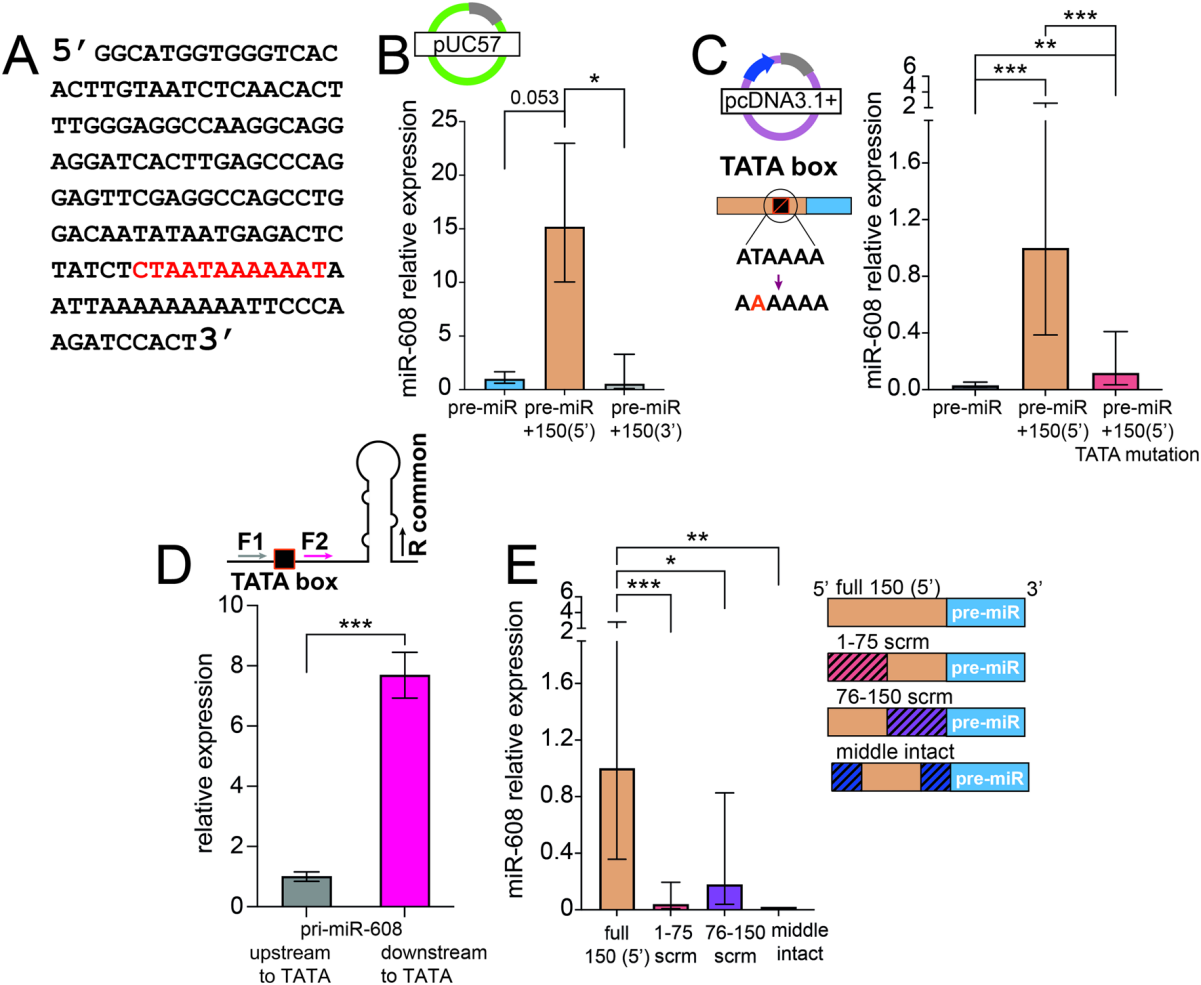
A TATA box enabling miR-608 expression is located in the 5′ 150 bases. **A** The 150 bases 5′ upstream to pre-miR-608 contain a TATA box at position 111 (red). **B** miR-608 levels in HEK293T cells transfected with pUC57, quantified by Taqman RT-qPCR, show that the TATA box drives expression from the promoter-less bacterial vector pUC57. **C**. miR-608 levels in HEK293T cells transfected with the mammalian vector pcDNA3.1 + containing the TATA box T-to-A point mutation are decreased as compared to non-mutated TATA box. **D** Higher levels of pri-miR-608 transcripts (expressed from pcDNA3.1 +) and measured by RT-qPCR using forward primers positioned upstream (F1) vs downstream (F2) to the predicted TATA box; the reverse primer (R) is common. **E** Scrambling parts of the 5′ 150 bases upstream to the pre-miR-608 sequence decreased the levels of mature miR-608, as determined by Taqman RT-qPCR quantification of miR-608 relative to the intact 150 base sequence and normalized to RNU6B. Hatching represents the scrambled area. Experiments were performed in duplicate or triplicate and in all panels *p < 0.05, **p < 0.01, ***p < 0.001. In panel D unpaired t-test, in all other panels one-way ANOVA with Tukey’s correction for multiple comparisons, bar-graph ± SD

As the 5′ 150-base sequence showed the most pronounced effect on miR-608 expression, we employed the “YAPP Eukaryotic Core Promoter Predictor” bioinformatic tool (http://www.bioinformatics.org/yapp/cgi-bin/yapp.cgi) to search for predicted promoter features and activator binding sites in this region. We identified a TATA box (CTAATAAAAAAT) at position 111 in the sequence (Fig. 2A) with high certainty (score of 0.89). This TATA box could potentially serve as a core promoter sufficient to drive miR-608 expression in a promoter-less vector. To test this assumption, we inserted pre-miR-608 and its 5′ 150 bases into the pUC57 bacterial vector which is devoid of a mammalian promoter. Supporting our prediction, when transfected into HEK293T and CT26.WT cells, the 5′ 150 bases elevated miR-608 levels about 15-fold over the pre-miR alone or the construct including the 3′ 150 bases. Thus, even without an external promoter both cell lines expressed miR-608 (Fig. 2B, Supp. Fig. 3B). To further assess the importance of the TATA box we mutated the thymine at position 5 to adenine and transfected HEK293T cells with this construct in pcDNA3.1 + , not pUC57, to better measure changes in expression from a base-line of 100-fold vs. 15-fold over pre-miR-608 (Figs. 1I vs. Fig. 2B). The levels of miR-608 were reduced by over 80%, confirming the importance of the TATA box in driving miR-608 expression (Fig. 2C).

The impact of the TATA core promoter on miR-608 expression was further tested by comparing pri-miR-608 levels that reflect transcription initiated from the CMV promoter (using a forward primer upstream to the TATA box) versus pri-miR-608 levels that reflect transcription initiated from the TATA box itself (using a forward primer downstream to the TATA box) (Fig. 2D, upper section). The level of pri-miR-608 transcripts initiated by the TATA core promoter were close to 8- fold higher than the levels initiated by the CMV promoter (Fig. 2D, lower section). In other words, roughly 85% of miR-608 is transcribed from the core promoter. This agrees with the results shown in Fig. 2C, whereby TATA box mutation reduced miR-608 by over 80%. Interestingly, partially scrambling stretches of 75 nucleotides in these 150 bases abolished (1–75) or greatly reduced (76–150) miR-608 expression, suggesting that expression may be affected by additional factors such as secondary structure or the location of important regulatory binding sites in this sequence (Fig. 2E, right and left panels).

### RPL24 is causally involved in mammalian miR biogenesis

**Fig. 3:**
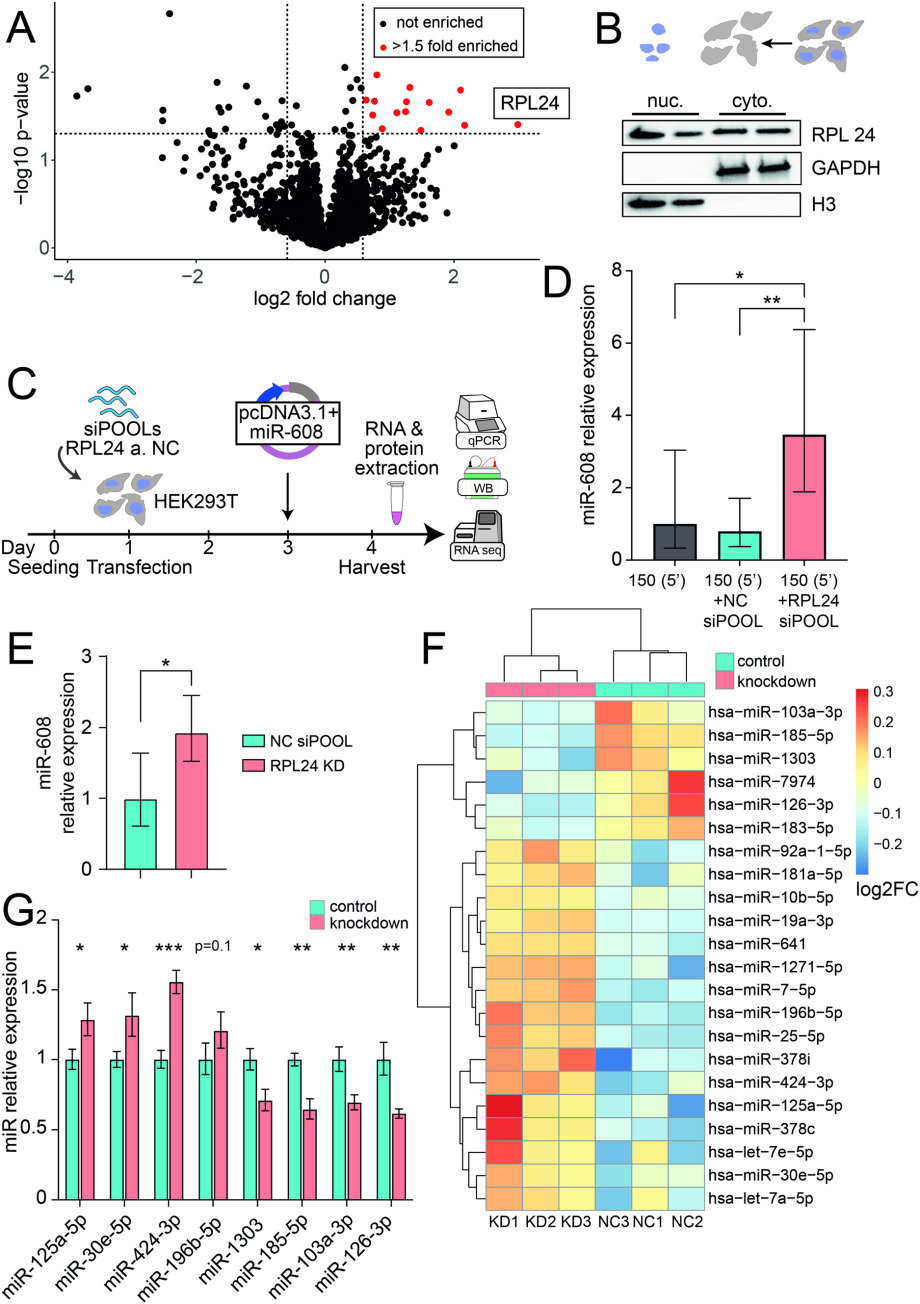
RPL24 KD alters the levels of diverse miRs in addition to miR-608. **A** Pull-down analysis: mass spectrometry identified 15 proteins bound to the 5′ 150 base-upstream sequence with p < 0.05 and enrichment > 1.5-fold (marked in red), the most enriched being RPL24 (*p* = 0.039, fold enrichment = 8). **B** Immunoblot of subcellular fractions identified RPL24 in both the nuclear and the cytoplasmic fractions. Fraction purity was validated with GAPDH, a cytoplasmic marker, and H3, a nuclear marker; each of which was localized solely to its expected compartment. **C** Experimental design: HEK293T cells were seeded, transfected with 50 nM of siPOOLs targeting RPL24 or a non-targeting negative control pool (NC) 24 h later, then transfected with pcDNA3.1 + containing miR-608 48 h later. RNA and proteins were extracted 24 h post-transfection. **D** RPL24 depletion in HEK293T cells elevates miR-608 levels. **E** RPL24 depletion in Caco2 cells shows the same effect. Quantification of miR-608 by Taqman RT-qPCR, with results normalized to RNU6B and relative to NC siPOOLs; for D, one-way ANOVA with Tukey’s correction for multiple comparisons ± SD. For E, unpaired t-test ± SD. **F** Heatmap showing the 22 DE miRs increased or decreased after RPL24 KD, analyzed by DESeq2, adjusted *p*-value < 0.05, Benjamini–Hochberg correction [27]. See Supp. Table 3 for full information on the DE miRs. **G** RT-qPCR validation confirming the RNA-seq results with data shown relative to the NC and normalized to SNORD47 and SNORD48; unpaired t-test for each miR ± SD, in panels **D**, E, **G**, *p < 0.05, **p < 0.01, ***p < 0.001

Numerous proteins impact the processing and expression levels of miRs [19, 20]. To identify proteins that interact with the 5′ 150 bases that control miR-608 levels we performed a pull-down assay in which we incubated a lysate of HEK293T cells with a biotinylated oligonucleotide comprised of the 5′ 150-base sequence. The proteins bound to the sequence were then isolated using streptavidin-coated magnetic beads (Supp. Fig. 4A) and identified by mass spectrometry (MS). This revealed the ribosomal protein RPL24 as the most enriched protein bound to the 5′ 150-base sequence (Fig. 3A). RPL24, a member of the L24E family of ribosomal proteins, is a component of the 60S ribosomal subunit and contributes to ribosome assembly and translational processes in the cytoplasm [21]. In *Arabidopsis thaliana*, a portion of STV1—the plant homolog of RPL24—localizes to the nucleus and affects the level of various miRs by binding a short 5′ sequence on the pri-miR and influencing their interaction with HYL1, a component of the plant microprocessor [22].

To investigate whether RPL24 can contribute to miR transcription and/or processing also in mammalian cells, we first sought to determine if RPL24 is present in the nuclear compartment, in which pri-miRs are transcribed and processed [5]. Using a standard fractionation protocol in HEK293T cells (see “Materials and Methods” Section) we confirmed that RPL24 is found not only in the cytoplasmic fraction as expected, but also in the nuclear fraction (Fig. 3B). To determine if this location indicates a possible role for RPL24 in regulating miR-608 expression in human cells we used a mixture of numerous low concentration siRNAs targeting RPL24 (siPOOLs) to knock-down (KD) RPL24 in HEK293T cells (Fig. 3C and Supp. Fig. 4B–C). The levels of mature miR-608 (Fig. 3D) were increased over threefold as compared to negative control (NC) siPOOLs, suggesting that RPL24 binding to the 5′ sequence of pri-miR-608 inhibits the expression of the mature miR-608 in mammalian cells. To verify that RPL24 has an inhibitory effect on expression of endogenous miR-608, we repeated the KD experiment in human Caco2 cells, which express miR-608 natively. Here too, we observed upregulation of the miR following RPL24 KD, confirming the inhibitory role of RPL24 also on the expression of endogenous miR-608 (Fig. 3E).

To examine the global impact of RPL24 on miR biogenesis we performed a separate RPL24 KD experiment in HEK293T cells, followed by small RNA sequencing. We were able to identify 22 differentially expressed (DE) miRs, 16 of which were increased and 6 decreased when compared to NC (Fig. 3F, Supp. Table 3). As HEK293T cells do not express miR-608, it was not detected in the sequencing dataset. RT-qPCR-quantification validated the sequencing data and confirmed significant changes in the levels of 7 out of 12 tested DE miRs: miR-125a-5p, miR-30e-5p and miR-424-3p were upregulated whereas miR-1303, miR-185-5p, miR-103a-3p and miR-126-3p were downregulated after RPL24 KD (Fig. 3G). These data support the general involvement of RPL24 in miR biogenesis in mammalian cells. The targets of the above DE miRs were identified and KEGG pathway analysis (using DIANA Tools, [23]) detected a marked enrichment in cancer-related pathways (Supp. Fig. 4D). Thus, while the role of RPL24 in tumorigenesis has so far been attributed to its involvement in translation [24–26], our current findings suggest RPL24 regulation of a specific set of miRs as potentially contributing to various cancers.

### RPL24 interacts with DDX5 and inhibits pri-miR processing

**Fig. 4:**
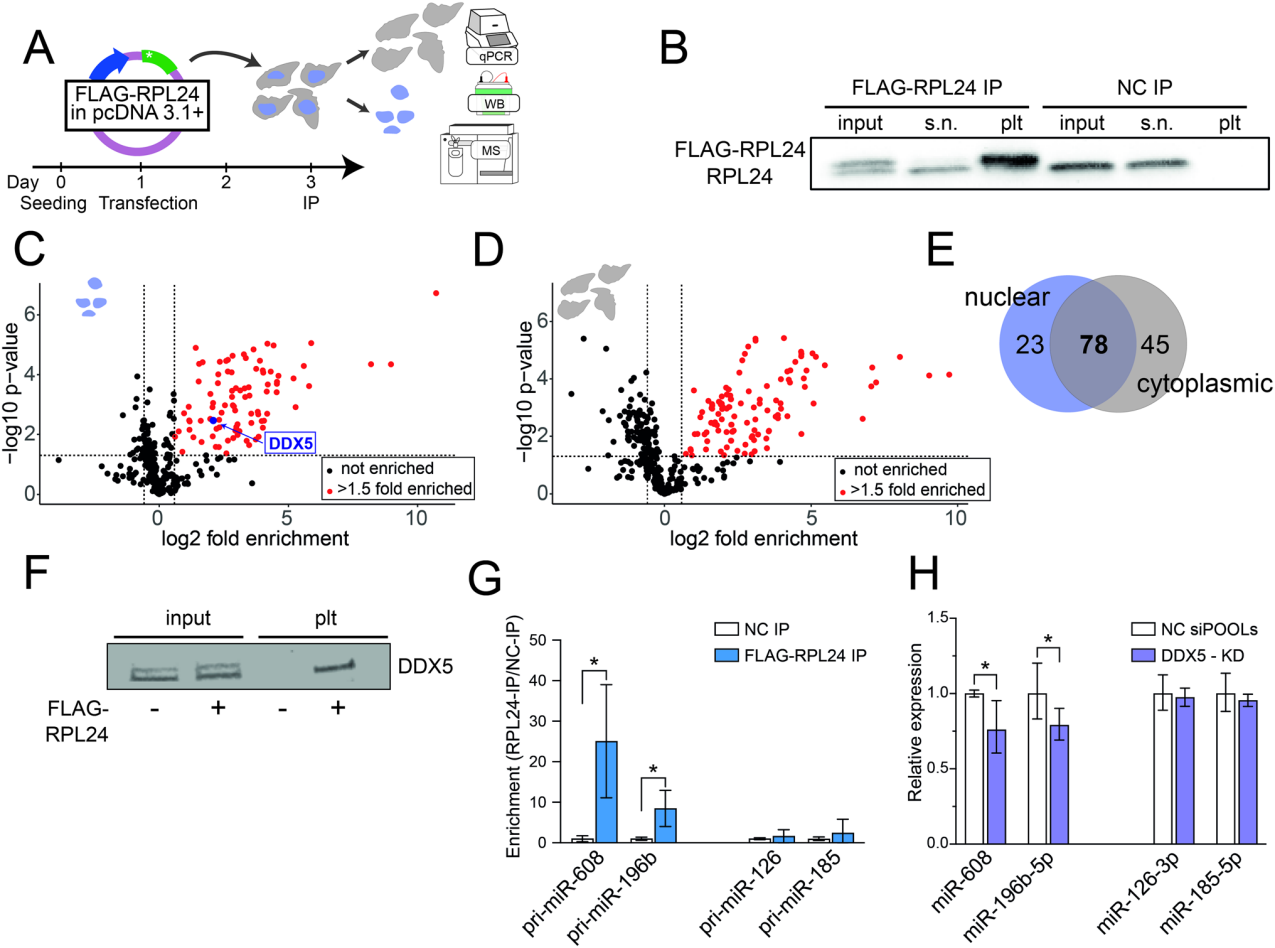
RPL24 binds pri-miRs and inhibits their processing through direct interaction with DDX5. **A** Experimental design: HEK293T cells were seeded and 24 h later transfected with pcDNA3.1 + vector containing FLAG-RPL24 or no insert as control. Cells were harvested 48 h after transfection, fractionated, and nuclear and cytoplasmic fractions immunoprecipitated separately with an antibody against the FLAG-tag, with subsequent MS analysis.** B** Immunoblot of nuclear fraction from cells transfected with a pcDNA3.1 + vector containing FLAG-RPL24 insert or empty pcDNA3.1 + vector as control, showing pull-down of the tagged-RPL24. 2.5% of input lysate, 2.5% of supernatant, and 20% of pellet were loaded per lane. Of the double band observed in the input lane, the upper and lower bands are the FLAG-RPL24 (~ 24kDa) and the endogenous RPL24 (~ 23kDa). **C**, **D** Volcano plot presenting the proteins enriched in FLAG-RPL24 IP compared to control, in the nuclear (**C**) and cytoplasmic (**D**) fractions. Red symbols denote proteins with *p* < 0.05 and enrichment > 1.5-fold and black symbols denote non-significant proteins.** E** Venn diagram showing proteins bound to RPL24 in cytoplasmic and nuclear fractions, with common and specific proteins. **F** Immunoblot showing the presence of DDX5 in RPL24 IP from the nuclear fraction of HEK293T cells transfected with pcDNA3.1 + vector containing ( +) vs lacking (−) a FLAG-RPL24 insert, confirming RPL24-DDX5 interaction. 2.5% of input lysate and 90% of pellet were loaded per lane. **G** RT-qPCR quantification of pri-miRs in nuclear pellet samples of FLAG-RPL24 IP. Pri-miR-608 and pri-miR-196b are enriched, but pri-miR-126 and pri-miR-185 are not. Each pellet sample was normalized to its corresponding input sample and fold enrichment is determined as FLAG-RPL24 IP/NC IP. **H** RT-qPCR quantification of miRs following DDX5 KD. miR-608 and miR-196b-5p are downregulated, but miR-126-3p and miR-185-5p levels are unchanged. For Panels G and H, experiments were performed in triplicate. Unpaired t-test, bar-graph ± SD, *p < 0.05

To further investigate the mechanism of RPL24 involvement in miR biogenesis we immunoprecipitated (IP) a FLAG-tagged RPL24 followed by MS analysis to identify interacting proteins. To identify specific interactions in the nucleus we fractionated the cells and then performed separate IPs for the nuclear and cytoplasmic fractions (Fig. 4A). Immuno-blotting confirmed the marked enrichment of FLAG-RPL24 in the pellet (Fig. 4B) and interacting partners were identified in a subsequent MS analysis. Over 100 proteins were found in each of the compartments (Fig. 4C–D, Supp. Tables 4 and 5), with over half of them shared (Fig. 4E). Predictably, most of these shared proteins were ribosomal proteins, which is expected, as ribosomal proteins synthesized in the cytoplasm are imported to the nucleus, assembled with rRNA into ribosomal subunits, then shipped back to the cytoplasm [28–30]. GO analysis [31] of the enriched proteins in both fractions revealed almost identical processes including 'cytoplasmic translation', 'ribosome biogenesis', 'ribonucleoprotein complex biogenesis', 'rRNA processing' and 'ncRNA processing' (Supp. Fig. 5A–B). Importantly, the RNA helicase DDX5, a component of the microprocessor, was enriched only in the nuclear pull-down fraction (Fig. 4C, 4-fold, *p* = 0.003), suggesting interaction of RPL24 with this complex. Immunoblot analysis of FLAG-RPL24 IP in an independent experiment showed the presence of DDX5 in the nuclear fraction, confirming this interaction (Fig. 4F).

To affirm that RPL24 executes its inhibiting effect on miR levels by binding to their pri-miRs, we immunoprecipitated RPL24 and extracted RNA from the nuclear pellet fraction. We then quantified the levels of pri-miR-608 and pri-miR-196b, the two most statistically significant miRs elevated by RPL24 KD. Both were enriched, indicating direct interaction between the protein and these pri-miRs (Fig. 4G, Supp. Table 3). In contrast, the levels of pri-miR-126 and pri-miR-185, the two most statistically significant miRs decreased by RPL24 KD (Supp. table 3), did not reveal any enrichment in the IP pellet (Fig. 4G), indicating a non-direct effect of RPL24 on these pri-miRs. These findings suggest that direct interaction of RPL24 with pri-miRs can inhibit pri-miR processing.

To examine whether this occurs via interaction with DDX5 we quantified the levels of these four miRs following KD of DDX5 (Supp. Fig. 5C–D). We found decreased levels of miR-608 and miR-196b-5p (by ~ 20%), supporting direct DDX5 involvement in their processing. In contrast, miR-126-3p and miR-185-5p, shown to lack direct interaction with RPL24, were not affected by DDX5 KD (Fig. 4H). Together, these findings confirm that RPL24 binds to pri-miR-608 and pri-miR-196b and inhibits their processing by interaction with DDX5. However, those miRs that were downregulated following RPL24 KD did not interact with RPL24 in the nucleus and their processing did not depend on DDX5, therefore their levels are most likely modified by other mechanisms.

## Discussion

The primate-specific hsa-miR-608 was the initial focus of our study. This miR plays a role in the cholinergic blockade of inflammation [13], cholinergic tone and stress responses [14], with functional and clinical consequences in carriers of SNPs in both miR-608 and its target AChE. Our current findings identify novel mechanisms regulating miR-608 expression.

To explore the function of miR-608 and the mechanisms controlling its expression we created a transgenic mouse expressing miR-608 flanked by 250 nucleotide-long regions and integrated into the third intron of the mouse Sema4g gene. The KI mice showed no behavioral or inflammatory phenotype, and levels of predicted miR-608 targets were also unchanged compared to control mice, probably due to the low expression levels of miR-608 in various tissues. Interestingly, the levels of the host gene Sema4g transcript were upregulated in the KI mice. This may be the result of the insertion of miR-608 with its flanking sequences into the mouse genome, as transgene insertion can affect the expression of proximal genes [32]. Importantly, there was no correlation between Sema4g and miR-608 levels, implying that additional factors can regulate miR-608 expression in addition to its host gene promoter [11, 12].

Due to the insufficiency of the KI mouse model, we focused on studying miR-608 expression in cultured cells. This revealed that the human intronic flanking sequences are functionally involved in regulating its expression. Specifically, expression of miR-608 flanked by serially shortened regions from its human genome location identified a 150 nucleotide-long sequence 5′ to pre-miR-608 which elevated miR levels by 100-fold. Moreover, we identified a TATA box within this sequence that acts as a core promoter to induce miR-608 expression independently of its host gene promoter. This is compatible with reports that intronic miRs that are co-expressed from the host promoter show high evolutionary conservation [33]. Nevertheless, independent expression of newer miRs such as the primate-specific miR-608 may confer tighter regulation and availability to function regardless of their host gene expression.

RPL24 is a key component of the ribosomal 60S subunit that is common to diverse organisms from archaea to eukaryotes. It operates as a translation factor which incorporates into the large ribosomal subunit and facilitates interaction between the large and small ribosomal subunits [21]. In mice, homozygous Rpl24 deficiency is lethal and heterozygous mice are viable but develop various abnormalities [34]. In *Arabidopsis thaliana*, mutated plants lacking the RPL24 homolog short valve 1 (STV1) demonstrate altered levels of several miRs compared to WT plants. STV1 localizes to the nucleus where it binds a short 5′ sequence of pri-miRs and promotes their processing by facilitating their interaction with the HYPONASTIC LEAVES 1 (HYL1) protein, a component of the plant miRNA processing complex [22].

Our immunoprecipitation experiments identified the involvement of RPL24 in regulating mammalian, and more specifically, primate miR processing. We have also shown for the first time that RPL24 is found not only in the cytoplasm but also in the nucleus of mammalian cells. In this cellular compartment it binds a 150-nucleotide-long sequence upstream to pre-miR-608 and inhibits miR-608 expression. Small RNA-sequencing following RPL24 depletion revealed altered levels of 22 miRs, suggesting that, as in plants, RPL24 is actively involved in fine-tuning the levels of numerous miRs. Intriguingly, some eukaryotic ribosomal proteins (e. g. RPL4, RPS4, 6, 7, 9, 14 and 14) are imported from the cytoplasm to the nucleolus where they participate in the assembly of ribosomal subunits that are then exported to the cytoplasm [28, 29]. Other ribosomal proteins have extra-ribosomal functions, including RPL11 which binds the MYC protein and inhibits the transcriptional activation of MYC-targeted genes [35] and RPS3 which can act as a caspase-dependent inducer of apoptosis and a DNA repair endonuclease [36, 37]. However, to our knowledge, none of these ribosomal proteins were shown to participate in pri-miR processing in mammalian cells.

The RPL24 immunoprecipitation experiments also provided a deeper understanding of the mechanism by which nuclear RPL24 executes its inhibiting effect on pri-miR processing. We identified a direct interaction between RPL24 and a component of the microprocessor, DDX5. In *Arabidopsis*, in contrast, STV1 was not found to interact directly with the plant microprocessor [22]. Thus, our results suggest an evolutionary conserved role for RPL24 in pri-miR processing, though its precise mechanism in primates may differ from that of plants.

In our RPL24 pull-down experiments we also identified enriched pri-miRs of those RPL24-inhibited miRs (miR-608 and miR-196b-5p, whose levels were increased upon RPL24 KD).We conclude that RPL24 inhibition of miR expression occurs upon its binding to pri-miRs in the nucleus. Furthermore, the levels of these miRs were downregulated following DDX5 KD, confirming that DDX5 promotes their processing. Together this suggests that RPL24 inhibition is executed by binding both the pri-miRs and DDX5, resulting in an interfered processing of these miRs. In contrast, primary sequences of miRs that were elevated following RPL24 depletion (such as miR-126-3p and miR-185-5p) were not bound by RPL24 and are not dependent on DDX5-mediated processing. Therefore, changes in their levels could be attributed to other mechanisms such as translational repression. Correspondingly, three of these miRs (miR-126, miR-185, and miR-103a) are significantly downregulated by depletion of another ribosomal protein, RPS15 [38]. Interestingly, our IP results show that RPS15 interacts with RPL24 in the cytoplasm. Thus, in the cytoplasm RPL24 depletion might indirectly affect miR levels due to its interaction with other ribosomal proteins and the general effect of translational repression. In comparison, in *Arabidopsis*, STV1 also indirectly influences the transcription of miRs by altering the occupancy of Pol II at their promoters and by affecting the levels of transcription factors regulating miR transcription [22]. However, RPL24 involvement in transcription was not tested in our study and calls for future research and ChiP tests, as it may shed light on additional mechanisms by which RPL24 contributes to mammalian miR biogenesis. In addition, given that SNPs in the miR-608 gene result in altered reactions to stress and inflammation [16], further examining of RPL24 regulation of miR-608 in various genotypes is called for, as it may offer new insights into RPL24 involvement in these processes.

Taken together, we have identified a novel evolutionarily conserved role for RPL24 in mammalian miR biogenesis, and showed that a portion of RPL24 is located in the nucleus, where it binds pri-miRs and suppresses their expression through direct interaction with the microprocessor. Our findings characterize a pan-mammalian extra-ribosomal role of RPL24 in miR biogenesis and reveal potential routes explaining the physiological impact of this phenomenon in primates.

## Materials and methods

### miR-608 knock-in mice

“Humanized” C57BL/6 miR-608 KI transgenic mice were produced in the EMBL Mouse Genomic Center, Monterotondo, Italy. Pre-hsa-miR-608 was inserted into the third intron of the mouse Semaphorin-4G gene (Sema4G) along with 250 endogenous flanking bases on each side. The targeting vector was introduced into R1 embryonic stem (ES) cells by standard methods. Resistant ES cell colonies were screened by Southern blotting, confirmed by PCR and cells containing the modified gene were used to generate chimeric mice. One founder mouse which gave germ line transmission was bred and mice were backcrossed for at least 8 generations to minimize genetic background heterogeneity. Brain tissue from medial prefrontal cortex, hippocampus, hypothalamus, and cerebellum and peripheral tissue from liver, kidney, heart, and intestine, were obtained from wild-type control and transgenic mice, both female and male.

### miR-608 constructs for cell culture

miR-608 constructs were inserted into the second intron of the human hemoglobin subunit beta (HBB) gene and cloned into the mammalian vector pcDNA3.1 + or into the bacterial vector pUC57.

### Cell culture

Human embryonic kidney cells (HEK293T, ATTC CRL-3216), human CACO-2 cells (Caco2, ATTC HTB-37™) and mouse colon carcinoma cells (CT26.WT, ATTC CRL-2638) were grown under standard conditions (see Supp. Materials and Methods) and guaranteed to be free of mycoplasma. Transfection was performed using Polyethylenimine (PEI) in HEK293T cells, HiPerFect transfection reagent (Qiagen, 301705) in Caco2 cells, and FuGENE™ HD Transfection Reagent (Promega, E2311) in CT26.WT cells. Cells were harvested 24 or 48 h post-transfection.

### RNA extraction

RNA was extracted using the miRNeasy Mini Kit (Qiagen, 217004) according to the manufacturer's protocol, followed by RNA concentration determination (NanoDrop 2000, Thermo Scientific) and standard gel electrophoresis.

### RT-qPCR

Synthesis of cDNA from mRNA and qPCR were done using Quantabio reagents and human-specific primers. Synthesis of cDNA from microRNA and qPCR were done using either Quantabio or TaqMan™ (Thermo Fisher Scientific) reagents. The CFX384 Touch Real-Time PCR System (Bio-Rad) was used for quantification and the CFX Maestro software (Bio-Rad v4.1.2433.1219) for analysis. Data is presented as relative expression (ΔΔCt) normalized to housekeeping genes and plotted as geometric mean ± geometric SD in GraphPad Prism 8.0 (GraphPad Prism Software) (See Supp. Materials and Methods).

### Core promoter

The “YAPP Eukaryotic Core Promoter Predictor” bioinformatic tool (http://www.bioinformatics.org/yapp/cgi-bin/yapp.cgi) was used to analyze the 150 bases 5′ upstream to pre-miR-608 and to confirm that the predicted TATA box could be abolished by inserting a point mutation. The TATA box was mutated by a thymine-to-adenine at position 115, using the QuikChange II site-directed mutagenesis kit (Agilent, 200521).

### Oligonucleotide pull-down assay

HEK293T cells were seeded in 100 mm plates, lysed 24 h later, and incubated with a 5′-biotinylated ssDNA oligonucleotide (sequence identical to the 150 bases upstream to pre-miR-608). Samples were incubated in a Thermo-shaker and streptavidin beads used to retrieve the oligonucleotide together with interacting proteins (for MS analysis) and RNA (for RT-qPCR or RNA-seq) (See Supp. Materials and Methods).

### RPL24 immunoprecipitation

HEK293T cells were seeded in 150 mm plates and transfected 24 h later with pcDNA3.1 + containing an insert of RPL24 labeled with C-terminal *Flag*^*®*^*-tag*)or no insert as control( and miR-608 plasmid. 48 h later cells were lysed in buffer containing 0.1% Triton X-100 and fractionated as above. Protein concentrations were determined and both fractions were incubated with Anti-FLAG^*®*^ M2 Magnetic Beads (Merck, M8823). Beads were washed once, then each sample was split for MS, immunoblotting, and RNA extraction. All samples were then washed three additional times (MS samples in detergent- and glycerol-free buffer, see supp Materials and Methods).

### Mass spectrometry

The bead-bound immunoprecipitated samples were denatured with 8M urea, treated with iodoacetamide, and trypsinized. Peptides were acidified with formic acid and desalted. MS/MS was performed on a Q Exactive™ Plus mass spectrometer coupled to a Dionex UltiMate 3000 system with peptides separated over a non-linear gradient of acetonitrile on a reverse phase C18 column. Data were acquired using Xcalibur™ software (all Thermo Fisher Scientific) and processed using the MaxQuant computational platform against a human reference proteome (UniProt UP000005640). Peptides with a length of at least seven amino acids were analyzed with FDR set at 1%. Relative protein quantification was determined using the label-free quantification algorithm. Statistical analysis was performed using the Perseus statistical package [39] with default software parameters for all statistical computations (See Supp. Materials and Methods).

### Subcellular fractionation

HEK293T cells were seeded in 6-well plates, harvested 24 h later, and lysed in buffer containing 0.1% Triton X-100. Nuclei were pelleted by centrifugation, supernatant containing the cytoplasmic fraction was collected and the pelleted nuclei lysed. Nuclear and cytoplasmic fractions were further clarified by high-speed centrifugation (See Supp. Materials and Methods).

### Immunoblots

Protein concentrations were determined using the Bradford (Merck, B6916) or Lowry assay (DC Protein Assay, Bio-Rad, 5000113) and 5 µg/sample was loaded onto 4–15% gradient polyacrylamide gels (Mini-PROTEAN TGX Gels, Bio-Rad, 4561083), transferred (Bio-Rad, Trans-Blot Turbo Transfer System) to nitrocellulose membranes (Bio-Rad, 1704158) and probed with antibodies against RPL24 (Proteintech, 17082–1-AP, 1:1000), B-Actin (Santa Cruz, sc-47778, 1:1000), A-Tubulin (Merck, T5168, 1:1000), GAPDH (Cell Signaling Technology, #2118, 1:1000), Histone H3 (abcam, ab1791, 1:1000) and DDX5 (Proteintech, 10804–1-AP, 1:700).

### RPL24 knock-down

HEK293T cells were seeded and 24 h later transfected with 50 nM of siPOOLs targeting RPL24 or non-targeting siPOOLs as control (ON-TARGETplus siRNA, Horizon, Perkin Elmer) using HiPerFect transfection reagent (301705, Qiagen). 48 h later cells were transfected with miR-608 plasmids using PEI. After an additional 24 h cells were harvested and RNA and protein extracted (See Supp. Materials and Methods). Caco2 cells were seeded and 24 h later transfected with 50 nM of siPOOLs targeting RPL24 or non-targeting siPOOLs as control. 48 h later cells were harvested and RNA extracted.

### DDX5 knock-down

HEK293T cells were seeded and 24 h later transfected with 50 nM of siPOOLs targeting DDX5 or non-targeting siPOOLs as control (ON-TARGETplus siRNA, Horizon, Perkin Elmer) using HiPerFect transfection reagent (301705, Qiagen). 24 h later cells were transfected with miR-608 plasmids. After an additional 24 h cells were harvested and RNA and protein extracted (See Supp. Materials and Methods).

### Small RNA sequencing

RNA was extracted from HEK293T cells as described above and RIN determined for all samples (RIN = 10 for all, Bioanalyzer 6000, Agilent). Libraries were constructed from 800 ng total RNA (NEBNext Multiplex Small RNA library prep set for Illumina, New England Biolabs, NEB-E7560S) and the small RNA fraction was sequenced on the NextSeq 500 System (Illumina) at the Center for Genomic Technologies Facility, the Hebrew University of Jerusalem.

### Analysis of small RNA sequences

Quality control parameters in the HEK293T (above) dataset were checked using FastQC (http://www.bioinformatics.babraham.ac.uk/projects/fastqc/). Reads were further trimmed and filtered using Flexbar (version 0.11.9 [40]). HEK293T sequences were aligned using miRExpress 2.1.4 [41] to miRBase version 21 for microRNAs. Raw files and metadata are available at the NCBI GEO database (accession number GSE224338). Differential expression analysis was performed in R version 4.0.2 using DESeq2 [27].

### Supplementary Information

Below is the link to the electronic supplementary material.

Supplementary file1 (DOCX 15609 KB)

Supplementary file2 (XLSX 39 KB)

Supplementary file3 (XLSX 20 KB)

Supplementary file4 (XLSX 20 KB)

Supplementary file5 (XLSX 29 KB)

## Data Availability

The original gene expression data (FASTQ files, metadata and tables of raw counts) from the RPL24 knockdown experiments in HEK293T cells are available at the NCBI GEO database under the accession number GSE224338. All other relevant datasets have been included as supplementary files to the manuscript and the original files and code are available upon request from corresponding authors (katarzyna.winek@mail.huji.ac.il and hermona.soreq@mail.huji.ac.il).
