# Supplementary material for: Ribosomal protein L24 mediates mammalian microRNA processing in an evolutionarily conserved manner
Source: Cell Mol Life Sci. 2024 Jan 23;81(1):55. doi: 10.1007/s00018-023-05088-w (PMC10805976; doi:10.1007/s00018-023-05088-w)
Supplement: Supplementary file 1 — Supplementary file1 (DOCX 15609 KB) [file 18_2023_5088_MOESM1_ESM.docx]

**Supplementary material, in order of presentation in the paper:**

1. Supp. Table 1: miR-608 predicted targets in human (submitted as a separate file)
2. Supp. Table 2: miR-608 predicted targets in mouse (submitted as a separate file)
3. Supp. Figure 1: Characterization of miR-608 KI mice
4. Supp. Figure 2: miR-608 targets and Sema4g in the KI mice
5. Supp. Figure 3: Basal and transfected miR-608 expression
6. Supp. Figure 4: RPL24 detection and knockdown
7. Supp. Table 3: Differentially expressed miRs identified following RPL24 knockdown
8. Supp. Table 4: Enriched proteins following FLAG-RPL24 IP in the nuclear fraction (submitted as a separate file)
9. Supp. Table 5: Enriched proteins following FLAG-RPL24 IP in the cytoplasmic fraction (submitted as a separate file)
10. Supp. Figure 5: GO analysis of RPL24 nuclear and cytoplasmic partners and validation of DDX5 knockdown
11. Supp. Table 6: qPCR primers used for quantification of mRNA targets
12. Supplementary Materials and Methods


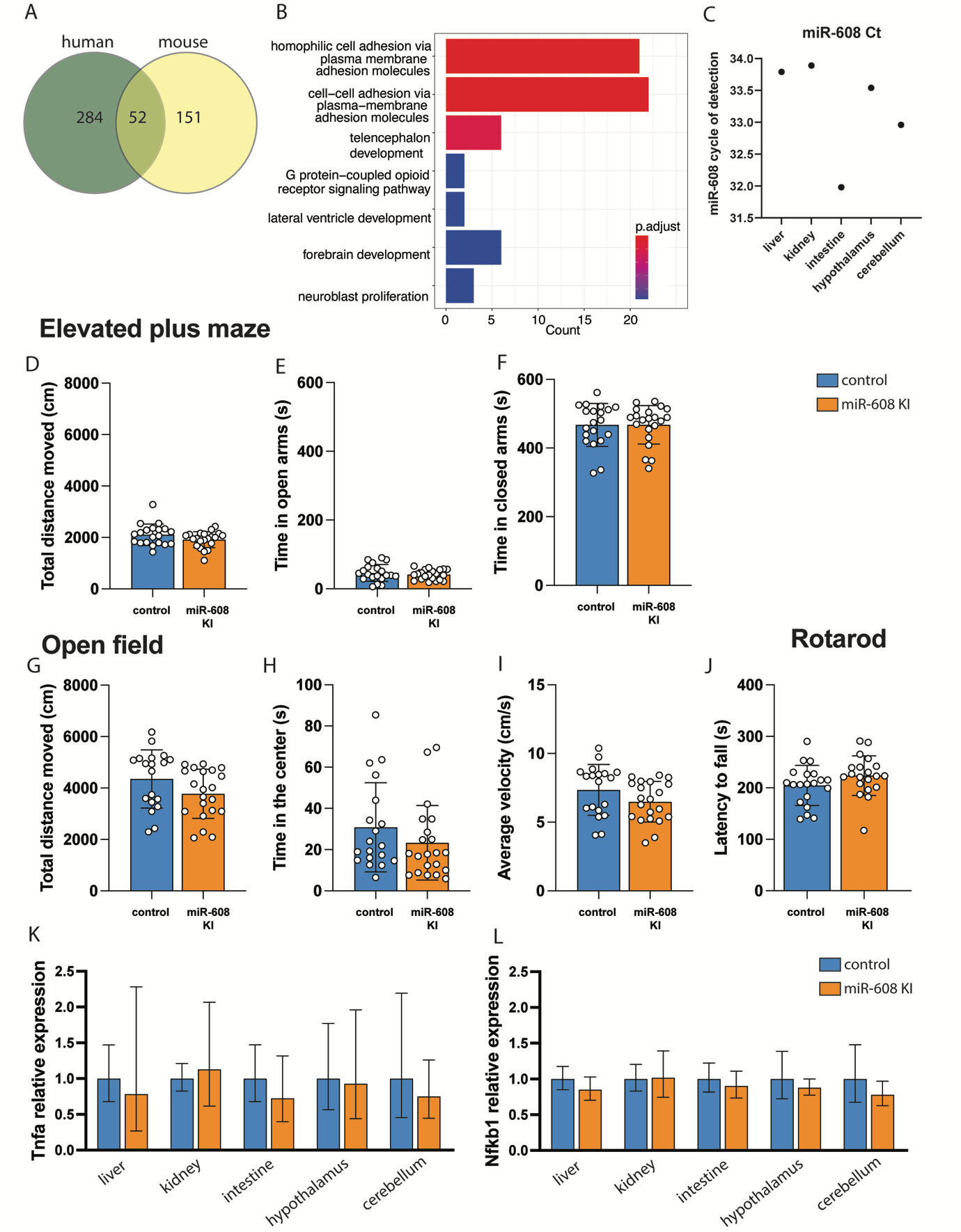


**Supp. Figure 1: Characterization of miR-608 KI mice**

**A.** Venn diagram of miR-608 predicted targets; 336 predicted targets in human (cumulative weighed context++ score≥ 0.7), 203 in mouse, 52 common to both. **B.** GO analysis of enriched processes of the 52 genes predicted to be targeted by miR-608 in both human and mouse. **C.** Average cycle of detection (not normalized to housekeeping gene) of miR-608 in the different miR-608-expressing tissues of the KI mice, n=10 for each tissue. **D-F.** Elevated plus maze test was conducted using a standard protocol [1]. **D.** Total distance moved; **E.** Time spent in open arms; **F.** Time spent in closed arms. All parameters were similar between miR-608 KI and control (non-transgenic) mice. **G-I.** Open field test was conducted using a standard protocol [2]. **G.** total distance moved; **H.** Time spent in center; **I.** Average velocity. No differences were detected between miR-608 KI and control mice. **J.** Rotarod test was conducted using a standard protocol [3]. The latency to fall was similar between miR-608 KI and control mice. In all cases n=17-21 mice per group. **K.** Tnfa and **L.** Nfkb1 expression levels in the brain and peripheral tissues of miR-608 KI mice, determined by RT-qPCR and normalized to ACTB, n=9-10 per group. Unpaired t-test was used for all comparisons.


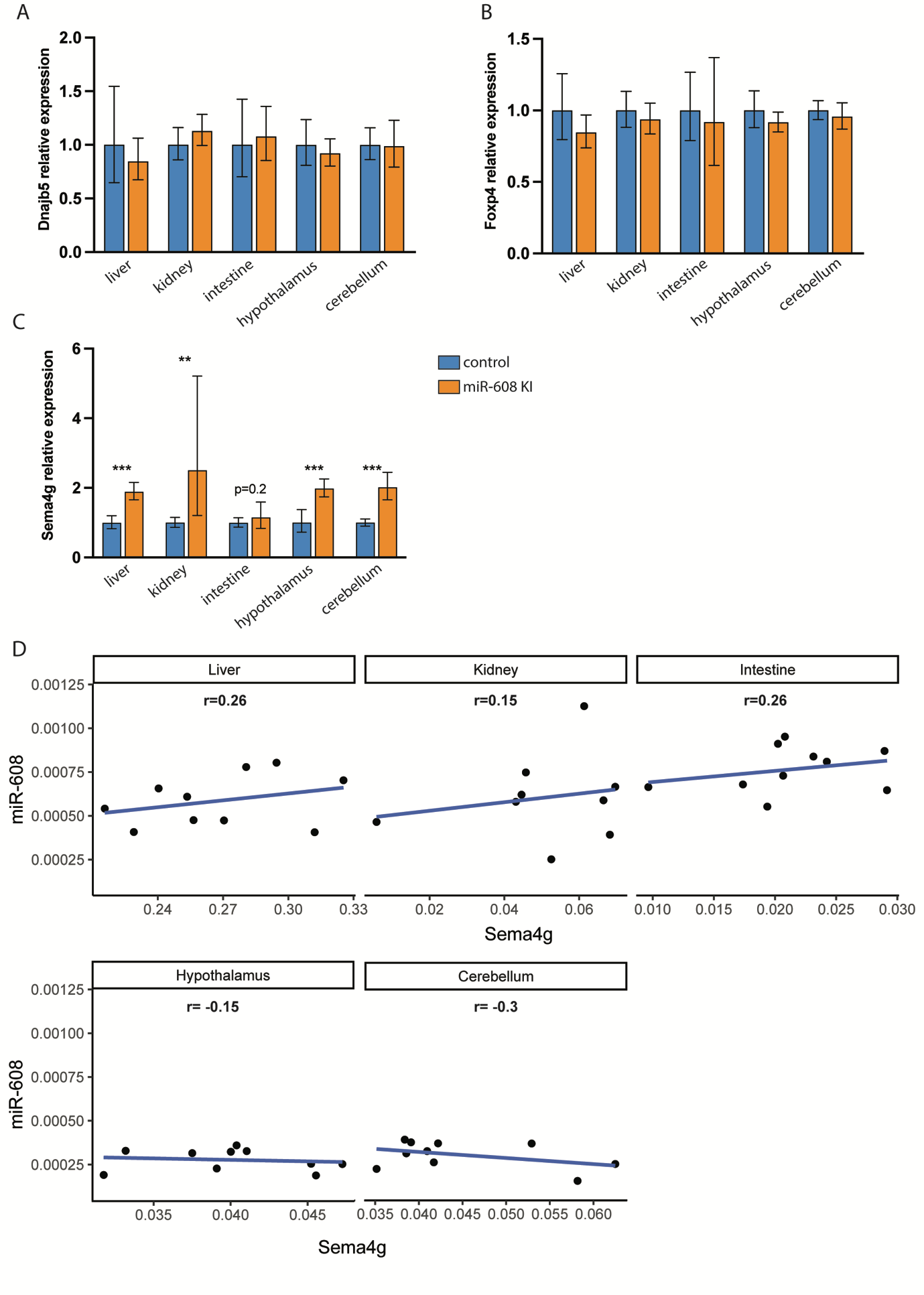


**Supp. Figure 2: miR-608 targets and Sema4g in the KI mice**

**A.** Dnajb5 mRNA levels in the brain and peripheral tissues of miR-608 KI mice. **B.** Foxp5 levels in the above tissues. **C.** Sema4g levels in the above tissue. All were quantified by RT-qPCR and normalized to ACTB, n=8-10 per group, unpaired t-test, bar-graph ± SD, * p <0.05, ** p <0.01, *** p <0.001. **D.** Spearman's correlation test between Sema4g levels (normalized to ACTB) and miR-608 levels (normalized to snoRNA135) in each of the miR-608-expressing tissues.


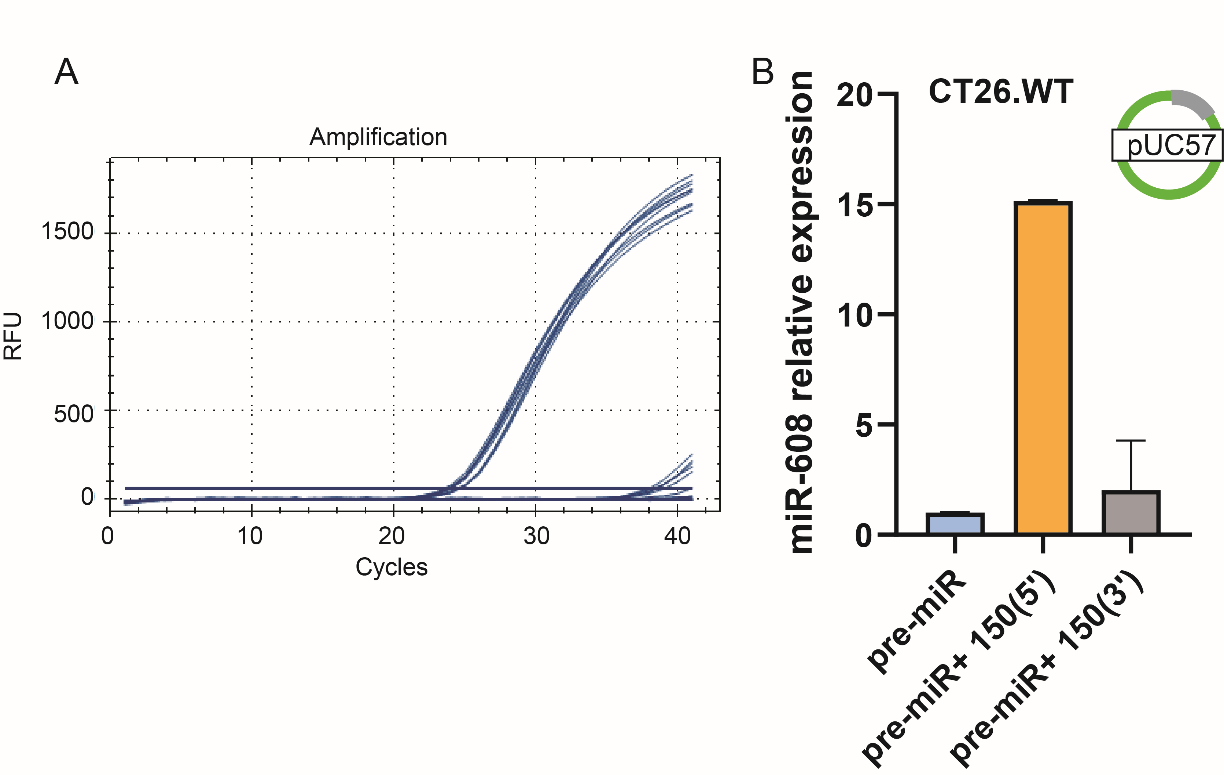


**Supp. Figure 3: Basal and transfected miR-608 expression**

**A.** HEK293T cells do not express miR-608; it is detected only at Ct > 39 which is identical to the NRT control. The RNU6B normalizing gene is detected at Ct 26. Quantification by RT-qPCR, experiments were performed in duplicate or triplicate. **B.** The 5’ 150 bases sequence, inserted into the pUC57 bacterial vector, increased miR-608 levels by ~15-fold in mouse CT26.WT cells. Quantification by Taqman qPCR, normalized to snoRNA135; one-way ANOVA, bar-graph ± SD, p=0.09.


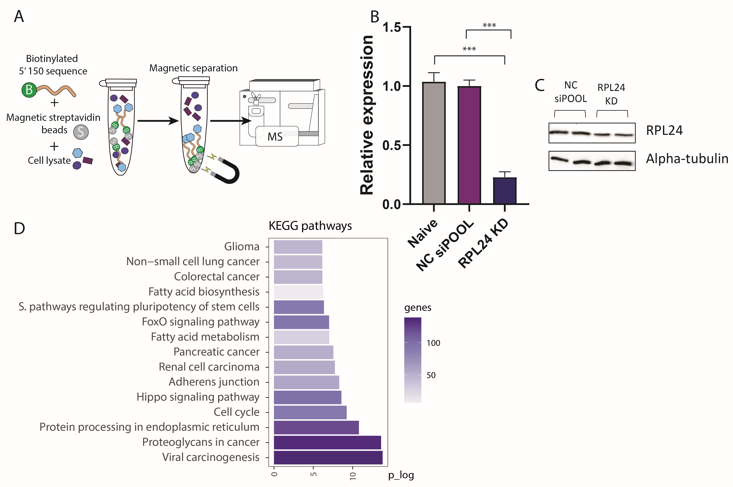


**Supp. Figure 4: RPL24 detection and knockdown**

**A.** Pulldown assay scheme: the biotinylated 5’ 150 sequence was incubated with HEK293T cell lysate, then isolated along with bound proteins using streptavidin-coated magnetic beads. The bead (pellet) samples were subjected to mass spectrometry to identify bound proteins. **B.** mRNA levels of RPL24, normalized to RPL19, in HEK293T cells transfected with siRNA (siPOOL) targeting RPL24 (RPL24 KD) or a non-targeting siPOOL control (NC). Reduction of ~80% in RPL24 mRNA levels is observed, confirming an efficient KD. Experiments were performed in triplicate, one-way ANOVA with Tukey’s correction for multiple comparisons, bar-graph ± SD, *** p <0.001. **C.** Western blot of RPL24 following KD compared to NC, normalized to alpha-tubulin. **D.** KEGG analysis showing the 15 most enriched pathways for targets of miRs regulated by RPL24.

|  | **baseMean** | **log2FoldChange** | **p value** | **p adj** |
| --- | --- | --- | --- | --- |
| hsa-miR-196b-5p | 2978.514867 | 0.388837084 | 1.44E-08 | 1.72E-06 |
| hsa-miR-7-5p | 36797.7353 | 0.331891923 | 1.14E-08 | 1.72E-06 |
| hsa-miR-378i | 1443.265975 | 0.44807567 | 6.46E-07 | 5.15E-05 |
| hsa-miR-125a-5p | 1565.015415 | 0.410845255 | 3.63E-05 | 0.002168504 |
| hsa-miR-185-5p | 1007.533487 | -0.336617352 | 5.63E-05 | 0.002487651 |
| hsa-miR-25-5p | 152.8001951 | 0.654926301 | 7.29E-05 | 0.002487651 |
| hsa-miR-424-3p | 362.6563998 | 0.479926819 | 6.53E-05 | 0.002487651 |
| hsa-miR-1271-5p | 226.2818677 | 0.563262702 | 0.000102583 | 0.003064673 |
| hsa-miR-378c | 397.5509962 | 0.480361268 | 0.000128387 | 0.003409394 |
| hsa-miR-126-3p | 856.0208311 | -0.346176952 | 0.000555804 | 0.013283713 |
| hsa-miR-19a-3p | 1000.288273 | 0.278416848 | 0.000741889 | 0.016119215 |
| hsa-miR-92a-1-5p | 95.6462453 | 0.670531668 | 0.001734386 | 0.034543188 |
| hsa-miR-7974 | 372.4094049 | -0.418385199 | 0.001960754 | 0.036047711 |
| hsa-miR-1303 | 361.4465324 | -0.377187148 | 0.002188845 | 0.037366708 |
| hsa-miR-10b-5p | 10905.75616 | 0.178957107 | 0.002543295 | 0.040523169 |
| hsa-miR-641 | 186.8236963 | 0.442512269 | 0.002981438 | 0.044535232 |
| hsa-let-7e-5p | 1332.731437 | 0.317044159 | 0.003525281 | 0.045040854 |
| hsa-miR-103a-3p | 7589.829015 | -0.223470153 | 0.003580654 | 0.045040854 |
| hsa-miR-30e-5p | 2883.119185 | 0.224342257 | 0.003218961 | 0.045040854 |
| hsa-miR-183-5p | 2192.05351 | -0.213272517 | 0.004111647 | 0.049134178 |
| hsa-let-7a-5p | 6258.931871 | 0.235730906 | 0.004345021 | 0.04935317 |
| hsa-miR-181a-5p | 497.061778 | 0.316356751 | 0.00454297 | 0.04935317 |

**Supp. Table 3:** **The differentially expressed miRs identified following RPL24 knockdown**.

Normalized counts, fold change vs control, and p values are presented.


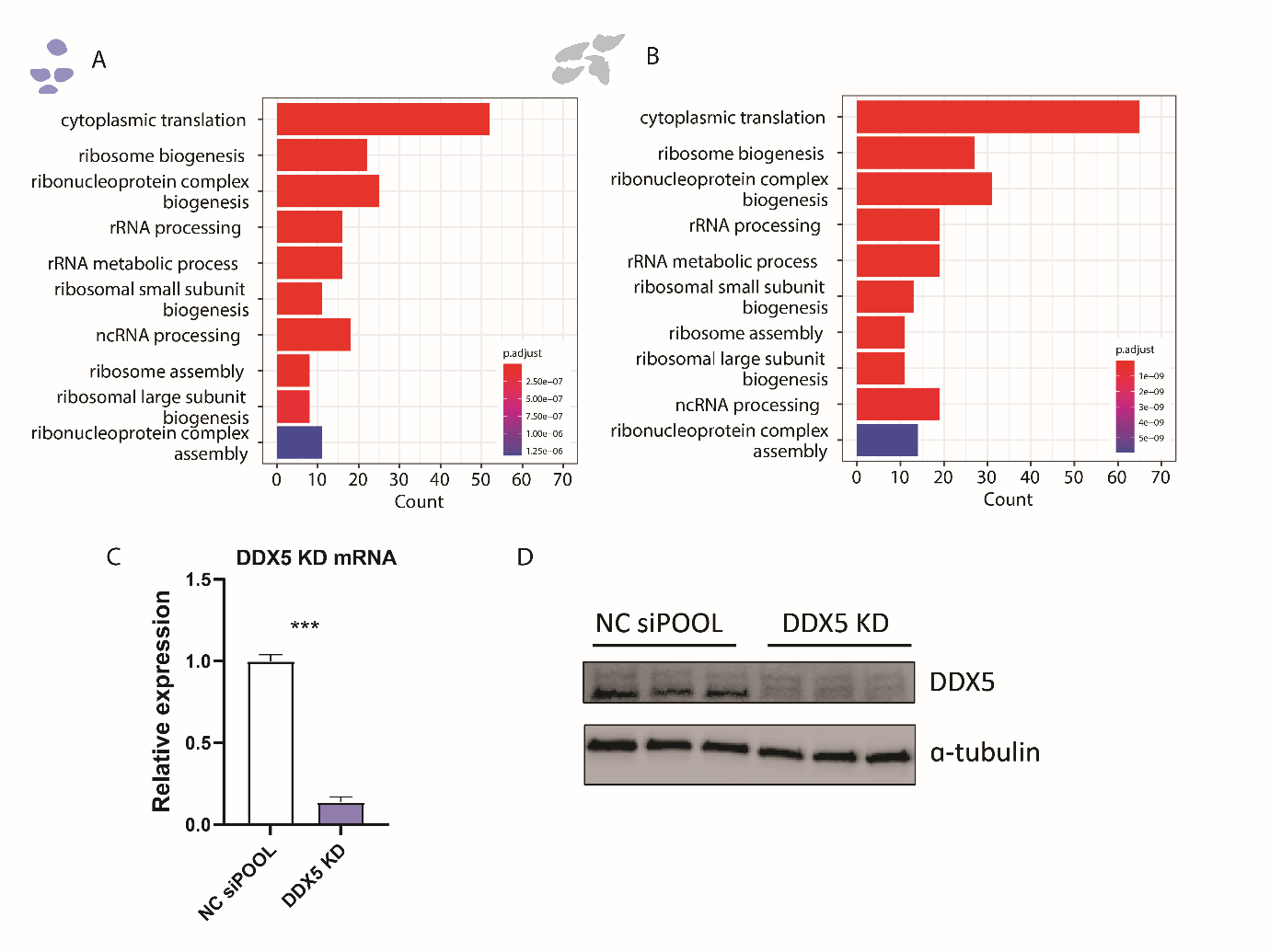


**Supp. Figure 5: GO analysis of RPL24 nuclear and cytoplasmic partners and validation of DDX5 knockdown**

**A.** GO analysis of the top 10 enriched processes of nuclear proteins interacting with RPL24. **B.** GO analysis of the cytoplasmic proteins. **C.** mRNA levels of DDX5, normalized to RPL19, in HEK293T cells transfected with siRNA (siPOOL) targeting DDX5 (DDX5 KD) or a non-targeting siPOOL control (NC). Reduction of ~85% in DDX5 mRNA levels is observed, confirming an efficient KD. Experiments were performed in triplicate, one-way ANOVA with Tukey’s correction for multiple comparisons, bar-graph ± SD, *** p <0.001. **D.** Western blot of DDX5 following KD compared to NC, normalized to alpha-tubulin.

| **Primer** | **Sequence (5’🡺3’)** |
| --- | --- |
| Mouse TNF-α Fwd | CCTGTAGCCCACGTCGTAG |
| Mouse TNF-α Rev | GGGAGTAGACAAGGTACAACCC |
| Mouse NFKB1 Fwd | ATGGCAGACGATGATCCCTAC |
| Mouse NFKB1 Rev | TGTTGACAGTGGTATTTCTGGTG |
| Mouse DNAJB5 Fwd | CCAGACAAGAACAAAGAGCCC |
| Mouse DNAJB5 Rev | CCTCACCATACTGGTCGTACA |
| Mouse SIX3 Fwd | CCGGAAGAGTTGTCCATGTTC |
| Mouse SIX3 Rev | CGACTCGTGTTTGTTGATGGC |
| Mouse FOXP4 Fwd | CACATCTCGGAGAGACAGCTC |
| Mouse FOXP4 Rev | CGTGTTCTGTGTTGAGGTGTT |
| Mouse ACTB Fwd | CCACACCCGCCACCAGTT |
| Mouse ACTB Rev | TACAGCCCGGGGAGCAT |
| Human RPL24 Fwd | CAAAAGAAAAGAACCCGCCGA |
| Human RPL24 Rev | TTCCTTAGCAGCCCTGATAGC |
| Human DDX5 Fwd | TCGCAGTACCAAAACAGGCA |
| Human DDX5 Rev | TACCCCTGGAACGACCTGAA |
| Human RPL19 Fwd | GCTCGATGCCGGAAAAACAC |
| Human RPL19 Rev | GCTGTACCCTTCCGCTTACC |
| Human Pri-miR-608 upstream to TATA box Fwd1 | GCCAGCCTGGACAATATAATG |
| Human Pri-miR-608 upstream to TATA box Rev | TCTACCTGTGCTGCACCAA |
| Human Pri-miR-608 downstream to TATA box Fwd2 | ACTGGGCCAAGGTGGGCC |
| Human Pri-miR-608 downstream to TATA box Rev | TCTACCTGTGCTGCACCAA |
| Human Pri-miR-196b Fwd | AGCCCTTCGCGGGCAGCAC |
| Human Pri-miR-196b Rev | CAACTGAAGTAATGAAGGCA |
| Human Pri-miR-185 Fwd | TGGGCAGTGGGCCTGGCTCGAG |
| Human Pri-miR-185 Rev | TGGGAGGGAAGGACCAGAGGA |
| Human Pri-miR-126 Fwd | CCAGCGCAGCATTCTGGAAG |
| Human Pri-miR-126 Rev | GTGGACGGCGCATTATTACTC |

**Supp. Table 6:** qPCR primers used for quantification of mRNA targets

**Supplementary Materials and Methods**

**Cell culture**

Cells were grown at 37oC, 5% CO2, in DMEM (Merck, D5671) (HEK293T), RPMI-1640 (Merck, R0883) (CT26.WT) or EMEM (Merck, M5650). All media were supplemented with FCS (10% final concentration, 04-127, Sartorius), L-glutamine (2mM final concentration, 03-020, Sartorius) and Penicillin-Streptomycin-Amphotericin (100 units/mL, 0.10 mg/mL, 0.25 µg/mL, final concentrations, respectively, 03-033, Sartorius). Cells were mycoplasma free (EZ-PCR™ Mycoplasma Detection Kit, Sartorius). For experiments, 75,000 cells/ml were plated and co-transfected 24 hours later with 500 ng miR-608 plasmid and in some cases (e. g. Figure 1) with 500 ng GFP plasmid for visualization. Transfection was performed using Polyethylenimine (PEI) in HEK293T cells, FuGENE™ HD Transfection Reagent (Promega, E2311) in CT26.WT cells, and HiPerFect transfection reagent (Qiagen, 301705) in Caco2 cells. Cells were harvested 48 hours post-transfection.

**RT-qPCR**

Synthesis of cDNA from mRNA and qPCR were done with qScript™ cDNA Synthesis Kit (Quantabio, 95047), PerfeCTa® SYBR® Green FastMix® (Quantabio, 95072), and human-specific primers (Suppl. Table 6). Synthesis of cDNA and qPCR for microRNAs were done with qScript™ microRNA cDNA Synthesis Kit (Quantabio, 95107), PerfeCTa® SYBR® Green FastMix® Low ROX (Quantabio, 95074), and human-specific primers (HSMIR-0196B-5P, HSMIR-0103A-3P, HSMIR-0185-5P, HSMIR-1303, HSMIR-0030E-5P, HSMIR-0424-3P, HSMIR-0007-5P, HSMIR-0019A-3P, HSMIR-0125A-5P, HSMIR-0010A-5P, HSLET-0007A-5P, all Quantabio). For miR-608, synthesis of cDNA from microRNA and qPCR were done with TaqMan™ MicroRNA Reverse Transcription Kit (Thermo Fisher Scientific, 4366596), TaqMan™ Universal PCR Master Mix (Thermo Fisher Scientific, 4304437) and TaqMan™ MicroRNA Assays (Thermo Fisher Scientific, 4427975; hsa-miR-608-001571, RNU6B-001093, snoRNA135-001230). Data is presented as relative normalized expression (ΔΔCt) with normalization as follows: all mRNAs to RPL19, all miRs except for miR-608 to SNORD47 and SNORD48, miR-608 to RNU6B in HEK293T and Caco2 cells and to snoRNA135 in CT26.WT cells.

**Oligonucleotide pull-down assay**

HEK293T cells were seeded in 100 mm plates (5x10^6^ cells/plate) and lysed 24 hours later in 600 µl buffer containing 10mM Tris-HCl pH 7.5, 500mM LiCl, 0.5% Triton X-100, 0.2% SDS, 0.2% sodium deoxycholate, Protease Inhibitor Cocktail (1:100, Cell Signaling Technology, 5871), and RNase inhibitor (1:100, NEB, M0314L). Lysates were then incubated on ice for 10 minutes and passed through a 27-gauge needle 5 times. 1200 µl of 1.5X hybridization buffer (15mM Tris-HCl pH 7.5, 7.5mM EDTA, 750mM LiCl, 0.75% Triton X-100, 0.3% SDS, 0.15% sodium deoxycholate, 3.75mM TCEP, protease and RNase inhibitors as above) were then added and samples were incubated 10 minutes on ice before clarification by high-speed centrifugation (10 minutes, 16,000g, 4^o^C). Protein concentrations were determined using the Lowry assay (DC Protein Assay, Bio-Rad, 5000113). 1500 µg protein/sample was incubated with 40 pmol of a 150-mer 5’-biotinylated oligonucleotide (sequence identical to the 5' 150 bases upstream to pre-miR-608, IDT, sequence below) for 4 hours, 37^o^C, 700 rpm, in a Thermo-shaker. 100 µl of Dynabeads® MyOne™ Streptavidin C (Thermo Fisher Scientific, Dy-65001) were added to each sample and incubation was continued for another hour. Beads were washed twice in buffer (10mM Tris-HCl pH 7.5, 5mM EDTA, 500mM LiCl, 0.5% Triton X-100, 0.2% SDS, 0.1% sodium deoxycholate, 2.5mM TCEP) and divided such that 50% of each sample was used for MS and 50% for RNA extraction. Samples used for RNA were washed three additional times in the above buffer, and samples used for MS were washed three additional times in buffer lacking detergent and glycerol.

Oligonucleotide sequence: 5'GGCATGGTGGGTCACACTTGTAATCTCAACACTTTGGGAGGCCAAGGCAGGAGGATCACTTGAGCCCAGGAGTTCGAGGCCAGCCTGGACAATATAATGAGACTCTATCTCTAATAAAAAATAATTAAAAAAAAATTCCCAAGATCCACT

**RPL24 immunoprecipitation**

HEK293T cells were seeded in 150mm plates (20 x10^6^ cells/plate) and transfected 24 hours later with 20 µg pcDNA3.1+ containing an insert of RPL24 labeled with C-terminal Flag*®-*tag )or no insert as control( together with 5 µg pcDNA3.1+ miR-608 plasmid. 48 hours later cells were lysed in 700 µl buffer containing 10 mM HEPES pH 7.9, 10 mM KCl, 1.5 mM MgCl2, 0.34 M sucrose, 10% glycerol, 1 mM DTT, and Protease Inhibitor Cocktail 1:100, with Triton X-100 then added to a final concentration of 0.1%, and incubated 5 minutes on ice. Nuclei were pelleted by centrifugation (4 minutes, 1300g, 4^o^C) and supernatant containing the cytoplasmic fraction was collected. Nuclei were suspended and lysed in 850 µl containing 30mM HEPES KOH pH 7.4, 100mM KAcetate, 2mM MgAcetate, 5% Glycerol, 0.5% IGEPAL CA-630, 1mM EGTA, 1mM DTT and Protease Inhibitor Cocktail and RNase inhibitor, both 1:100. Nuclear and cytoplasmic lysates were then rotated for 15 minutes at 4^o^C and clarified by high-speed centrifugation (15 minutes, 20,000g, 4^o^C). Protein concentrations were determined using Bradford assay (Merck, B6916) and 1200 µg protein of nuclear lysate and 1500 µg protein of cytoplasmic lysate were incubated with Anti-FLAG® M2 Magnetic Beads (Merck, M8823) for 4 hours with rotation at 4^o^C. Beads were washed once in buffer containing 30mM HEPES KOH Ph 7.4, 100mM KAcetate, 2mM MgAcetate, 5% Glycerol, 0.5% IGEPAL CA-630, 1mM EGTA, 1mM DTT, then divided such that 30% of each sample was used for MS, 10% for immunoblotting, and 60% for RNA extraction. Samples used for RNA and immunoblots were washed three additional times in above buffer, and samples used for MS were washed three additional times in the same buffer lacking detergent and glycerol.

**Mass spectrometry**

The immunoprecipitated samples designated for MS were washed three times in detergent- and glycerol-free buffer (30mM HEPES KOH Ph 7.4, 100mM KAcetate, 2mM MgAcetate, 1mM EGTA, 1mM DTT), the packed beads resuspended in 100 μl buffer (8M urea, 10 mM DTT, 25 mM Tris-HCl pH 8.0) and incubated for 30 minutes. Iodoacetamide (55 mM final concentration) was added and the beads incubated (30 minutes, dark, gentle agitation). The urea was diluted (8 volumes of 25 mM Tris-HCl pH 8.0) and 0.4 μg/ sample trypsin was added. Beads were incubated overnight at 37°C with gentle agitation and then peptides were acidified with formic acid (final concentration 0.38%) and desalted. MS was performed on a Q Exactive™ Plus mass spectrometer coupled on-line to a Dionex UltiMate™ 3000 system, with Xcalibur™ software for data acquisition (Thermo Fisher Scientific). Peptides (0.45 μg, estimated by O.D. 280 nm) were separated over an acetonitrile gradient (0 - 80%), flow rate 0.15-0.3 μl/min on a reverse phase 25-cm-long C18 column (75 μm ID, 2 μm, 100Å, PepMap RSLC) for 120 minutes. Survey scans (380–2,000 m/z, target value 3E6 charges, maximum ion injection times 50 ms) were acquired and followed by higher energy collisional dissociation-based fragmentation. A resolution of 70,000 was used for survey scans and up to 15 dynamically chosen most abundant precursor ions with “peptide preferred” profile were fragmented (isolation window 1.6 m/z). The MS/MS scans were acquired at a resolution of 17,500 (target value 1E5 charges, maximum ion injection times 120 ms). Dynamic exclusion was 60 sec. To avoid carryover, the column was washed with 80% acetonitrile, 0.1% formic acid for 25 min between samples. Data was processed using the MaxQuant computational platform. Peak lists were searched against the human reference proteome from UniProt (UP000005640) and allowed up to two mis-cleavages. Peptides with a length of at least seven amino acids were analyzed and the required FDR was set to 1% at the peptide and protein level. Relative protein quantification was determined using the label-free quantification (LFQ) algorithm. Statistical analysis was performed using the Perseus statistical package [4] with default software parameters used for all statistical computations.

**Subcellular fractionation**

HEK293T cells were seeded in 6-well plates (300,000 cells/well) and 24 hours later collected in 400 µl buffer containing 10 mM HEPES pH 7.9, 10 mM KCl, 1.5 mM MgCl2, 0.34 M sucrose, 10% glycerol, 1 mM DTT, Protease Inhibitor Cocktail 1:100 (Cell Signaling Technology, 5871), and then Triton X-100 was added to a final concentration of 0.1% and samples incubated for 5 minutes on ice. Nuclei were pelleted by centrifugation (4 minutes, 1300g, 4^o^C) and the supernatant containing the cytoplasmic fraction was collected. Nuclei were then lysed in 200 µl containing 3 mM EDTA, 0.2 mM EGTA, 1 mM DTT, and Protease Inhibitor Cocktail as above. Nuclear and cytoplasmic fractions were further clarified by high-speed centrifugation (20 minutes, 20,000g, 4^o^C) with supernatants collected for use.

**RPL24 knock-down**

HEK293T cells were seeded in 24-well plates for RNA (75,000 cells/well) and in 12-well plates for protein (150,000 cells/well) and transfected 24 hours later with 50 nM of siPOOLs targeting RPL24 or non-targeting siPOOLs as control (ON-TARGETplus siRNA, Horizon Discovery, PerkinElmer) using HiPerFect transfection reagent (Qiagen, 301705). 48 hours later cells were transfected with miR-608 pcDNA3.1+ plasmids. After an additional 24 hours (72h after siPOOL transfection) cells were harvested. For RNA extraction cells were collected in QIAzol. For protein extraction cells were washed twice in PBS and lysed in 140 µl of RIPA buffer (50mM Tris-HCl pH 7.5, 150mM NaCl, 0.5% sodium deoxycholate, 1% Triton X-100, 0.1% SDS, 1mM EDTA, with Protease Inhibitor Cocktail and Phosphatase Inhibitor Cocktail both diluted 1:100). Cells were incubated for 20 minutes on ice, collected, centrifuged (15 minutes, 20,000g, 4^o^C), and supernatant was centrifuged again as above. Supernatant was transferred to fresh tubes and protein concentration was determined by Lowry assay.

**DDX5 knock-down**

HEK293T cells were seeded in 12-well plates for RNA and protein (150,000 cells/well) and transfected 24 hours later with 50 nM of siPOOLs targeting DDX5 or non-targeting siPOOLs as control (ON-TARGETplus siRNA, Horizon Discovery, PerkinElmer) using HiPerFect transfection reagent (Qiagen, 301705). 24 hours later cells were transfected with miR-608 pcDNA3.1+ plasmids. After an additional 24 hours (48h after siPOOL transfection) cells were harvested. For RNA extraction cells were collected in QIAzol. For protein extraction cell fractionation was conducted as described above and protein concentration was determined by Bradford assay.

Supplementary references:

1. Rodgers, R.J. and A. Dalvi, *Anxiety, defence and the elevated plus-maze.* Neurosci Biobehav Rev, 1997. **21**(6): p. 801-10.

2. Prut, L. and C. Belzung, *The open field as a paradigm to measure the effects of drugs on anxiety-like behaviors: a review.* Eur J Pharmacol, 2003. **463**(1-3): p. 3-33.

3. Carter, R.J., J. Morton, and S.B. Dunnett, *Motor coordination and balance in rodents.* Curr Protoc Neurosci, 2001. **Chapter 8**: p. Unit 8 12.

4. Tyanova, S., et al., *The Perseus computational platform for comprehensive analysis of (prote)omics data.* Nat Methods, 2016. **13**(9): p. 731-40.
